# Knowledge of and Attitudes Toward Hearing Loss Among Primary Care Physicians in the Public Health Sector of Mauritius

**DOI:** 10.1055/s-0043-1770729

**Published:** 2023-10-06

**Authors:** Taslima Foondun, Lidia Pottas, Maggi Soer

**Affiliations:** 1 Department of Speech-Language Pathology and Audiology, Faculty of Humanities, University of Pretoria, Gauteng, South Africa; 2 Speech Therapy and Audiology Unit, Jawaharlal Nehru Hospital, Mauritius

**Keywords:** primary care physicians, public health sector, hearing loss, knowledge, attitudes

## Abstract

**Introduction** Primary care physicians are essential first points of contact for patients with hearing loss. Thus, knowledge of hearing loss and related aspects is essential to ensure the optimal management of individuals with suspected hearing loss.

**Objective** This study aimed to determine the knowledge of and attitudes toward hearing loss among primary care physicians in the public health sector in Mauritius.

**Methods** In this cross-sectional descriptive cohort study, 320 primary care physicians completed an online questionnaire adapted from previous questionnaires on knowledge of and attitudes toward hearing loss. Responses were analyzed using descriptive statistics and cross-sectional analyses.

**Results** Primary care physicians showed limited knowledge of hearing loss in areas such as early identification and intervention, professionals responsible for hearing assessments, and hearing tests used for assessing hearing sensitivity. However, the responses also showed positive attitudes toward hearing loss. Significant associations between knowledge of and attitudes toward hearing loss were obtained regarding the type of physician, length of practice, and department posted in. Ear, nose, and throat specialists, as well as pediatricians, demonstrated significantly higher scores for both knowledge of and positive attitudes toward hearing loss.

**Conclusions** The findings highlight a strong need for ongoing medical education to spread awareness about hearing loss among primary care physicians in the public health sector of Mauritius.

## Introduction

Hearing loss is one of the most common health concerns worldwide. 1 According to the World Health Organization (WHO), ∼466 million people globally experience disabling hearing loss, including 432 million (93%) adults and 34 million (7%) children. 2 By 2050, 2.45 billion people will have some degree of hearing loss. 3 If not adequately addressed, hearing loss can significantly influence the individual's communication, cognition, education, employment, and overall well-being. 4 Early identification and intervention for hearing loss can reduce or prevent these adverse consequences. 5

Primary care physicians (PCPs) and audiologists are often involved together in identifying and managing hearing loss in neonates, children, and adults. 6 PCPs function as gatekeepers who may refer patients with hearing loss to the appropriate department. 7 In 2020, a total of 4.5 million cases were treated in Mauritius' public health sector, which is primarily available to the Mauritian population. 8 Mauritius' public health sector is arranged according to a hierarchy of services such as primary, secondary, and tertiary and provides free health services, with the Ministry of Health and Wellness playing a significant role in the Mauritian welfare state. 8 PCPs in the Mauritian public health sector include general practitioners and medical interns who may guide patients and families through diagnostic and treatment procedures for hearing loss 9 ; ear, nose, and throat (ENT) specialists 10 ; pediatricians 11 ; and general medicine specialists who manage adults with vertigo or balance problems. 12

Several international studies have investigated the knowledge of and attitudes toward hearing loss among PCPs. These studies have demonstrated both poor and positive attitudes toward hearing loss, 13 14 vestibular disorders, and assessment procedures. 7 A lack of knowledge regarding the diagnosis and treatment of hearing loss, 13 hearing tests used to assess hearing sensitivity in children, 14 and risk factors of hearing loss 6 were also observed. To our knowledge, no published study has explored the knowledge of and attitudes toward hearing loss among PCPs in Mauritius.

The current study aimed to evaluate the knowledge of and attitudes toward hearing loss among PCPs employed in the public health sector of Mauritius. Within this public health system, appropriate channeling of patients, who might be at risk for hearing loss, to audiology departments is essential to ensure that these patients undergo the appropriate management. Thus, PCPs in the public health sector of Mauritius should have adequate knowledge of hearing loss to provide patients and families with the most current information, thereby facilitating appropriate and timely treatment. 14 Additionally, audiologists in Mauritius' public health sector should understand PCPs' knowledge of and attitudes toward hearing loss to ensure that they can provide appropriate support to PCPs.

## Subjects and Methods

A non-experimental, cross-sectional descriptive survey design was implemented using an online questionnaire via the hospital communication platforms. Participating PCPs provided informed consent, and anonymity was ensured. The study was approved by the Ministry of Health and Wellness, Mauritius, and by the Research Ethics Committee of the Faculty of Humanities, the University of Pretoria, South Africa [HUM004/0921].

### Study Population

PCPs were selected through non-probability purposive sampling from five regional hospitals in the public health sector of Mauritius. Only PCPs fluent in English were invited to participate. Using a 95% confidence interval and a margin of error of 5%, the minimum required sample size was computed as 309. 15 A total of 320 valid questionnaires was retrieved.

### Questionnaire

The current questionnaire was adapted from previous questionnaires 13 16 to ensure consistency with previous studies. The questionnaire was administered online using Google Forms and comprised of 16 questions organized into three sections Supplementary Appendix A . Section A (four statements) explored respondents' demographics, e.g., type of physician, practice setting, department, and years of practice. Section B focused on the PCPs' knowledge of hearing loss and consisted of two items evaluated on a five-point Likert scale ranging from “strongly disagree” to “strongly agree,” two multiple-choice questions, and two open-ended questions. Section C assessed the PCPs' attitudes toward hearing loss and focused on six statements that were presented on a Likert scale.

### Validation of the Study Tool

The survey was pilot tested for length, coherence, and consistency on 10 PCPs who were excluded from subsequent analyses. The questionnaire was modified to incorporate feedback suggestions. The questionnaire was internally consistent with Cronbach's α values of 0.894 and 0.906 for knowledge of hearing loss and attitudes toward hearing loss.

### Data Analysis

Quantitative data were analyzed using SPSS version 26 (IBM Corporation, Armonk, New York). Descriptive statistics were used to report the frequency of responses, and results are presented using tables and charts. The Shapiro–Wilk test showed that the data were not normally distributed. Nonparametric Mann-Whitney U and chi-square tests were used to determine significant differences in the knowledge of and attitudes toward hearing loss in relation to the type of physician, practice setting, number of years of practice, and department. Inferential analyses included correlation, simple regression, and cross-sectional analyses. The default significance level was 5%, but the results were reported according to the closest *p* -value. For the open-ended questions, thematic analysis was used to organize responses into themes, which are presented in terms of frequency.

## Results

### Demographic Information

Table 1 presents the demographic information of the 320 survey respondents. Most respondents were either general practitioners (43.8%) or medical interns (33.4%), with the majority of respondents (84.3%) practicing in hospitals. Regarding experience, 51.6% of respondents had practicing experience of <5 years, whereas 21.2% and 27.2% had been practicing for 5–9 and ≥10 years, respectively. While 21.9% of respondents worked in pediatric departments, 19.7% worked in casualty wards, and 18.7% worked in medical units. While 10.3% of the respondents worked in ENT departments, 11.3% were posted in unclassified departments, and 18.1% practiced in other departments.

**Table 1:** Demographic profile of the respondents ( *n* = 320)

Variables	Attributes	Frequency	Percentage
Type of physician	General practitioner ENT specialist Pediatrician Medical specialist Medical intern Other	141 16 27 21 107 9	**43.8** 5.0 8.4 6.6 33.4 2.8
Practice setting*	Area health center Hospital Community health center Medi-clinic Other	27 269 31 13 13	8.5 **84.3** 9.7 4.1 4.1
Number of years of practice	<5 5–9 ≥10	168 68 87	**51.6** 21.2 27.2
Current department	Pediatric Medical ENT Casualty Unsorted Other	70 60 33 63 36 58	**21.9** 18.7 10.3 19.7 11.3 18.1

Abbreviation: ENT, ear, nose, and throat.

* Multiple responses were possible.

### Knowledge of Hearing Loss

As shown in Table 2 , 35% of respondents either agreed that treatment exists to manage hearing loss or were not sure (M = 3.68). Similarly, 32–33% either agreed or were unsure that hearing loss could be identified at any given age (M = 3.66).

**Table 2:** Knowledge of hearing loss

	SD	D	N	A	SA	Mean
5. Treatment exists to manage hearing loss	3%	5%	35%	35%	22%	**3.68**
6. Hearing loss can be identified at any given age	3%	8%	33%	32%	24%	**3.68**
**Overall knowledge**	**3%**	**6%**	**34%**	**34%**	**23%**	**3.68**

Abbreviations: A, agree; D, disagree; N, neutral; SA, strongly agree; SD, strongly disagree.

To the multiple question asking respondents to identify the professionals responsible for hearing assessments, most respondents (42.0%) believed that hearing evaluations should be conducted by ENT specialists, whereas 37.0% reported that hearing assessments should be performed by audiologists. Among the remaining respondents, 10.6% and 8.4% stated that general practitioners and pediatricians should conduct hearing assessments, respectively. However, 2.0% of the respondents proposed other physicians, such as occupational health physicians.

A multiple-choice question (item 8) evaluated an important feature of hearing assessments, i.e., whether hearing tests are noninvasive. Three options were provided, with the correct answer being option (a) (noninvasive). More than half of the respondents (51.6%) indicated correctly that hearing assessments are noninvasive and can be performed at any age. According to 39.1% of the respondents, hearing assessments are reliable and precise when the central nervous system reaches maturation. The remaining 9.4% of respondents claimed that hearing assessments are invasive but provide accurate information at any age.

The open-set questions 9 and 10 asked physicians to list all hearing tests they knew to assess hearing sensitivity in children and adults, respectively. In children, otoacoustic emissions were the most popular answer (29.2% of responses), followed by auditory brainstem responses (27.5% of responses) and tuning forks (25.6% of responses). Immitance testing and speech audiometry were mentioned in only 4.6% and 3.3% of responses, respectively. Other responses, such as computed tomography, magnetic resonance imaging, electroencephalography, and whisper tests, were mentioned 5% of times, whereas 4.8% of respondents were unsure about any hearing assessments.

In adults, tuning fork tests (42.4% of responses) was the most popular answer, followed by pure tone audiometry (27.4% of responses) and other responses (7.3%), which included computed tomography, short increment sensitivity index measurements, whisper tests, and electroencephalography. Only 6.9% of respondents indicated auditory brainstem response measurements, and 6.7% of respondents indicated immittance testing. 3.9% of respondents mentioned speech audiometry. Otoacoustic emissions were noted in 2.7% of responses. 2.7% of respondents answered that they did not know of a single hearing assessment.

### Attitudes Toward Hearing Loss

As seen in Table 3 , most respondents strongly agreed that hearing loss is a condition that can become extremely debilitating (M = 4.25) and that hearing plays an important role in the acquisition of speech and language of a child (M = 4.40). Most respondents agreed that suspicion of hearing loss is a strong enough reason for referral to audiologists by an individual, parent, or caregiver (M = 4.13) and strongly agreed that hearing screening for newborns is essential (M = 4.23). Most respondents knew that, when necessary, hearing aids can be fitted to children of any age (M = 3.46). Finally, most respondents wished to receive additional information regarding hearing loss (M = 4.01).

**Table 3:** Attitudes toward hearing loss

	SD	D	N	A	SA	Mean
11. Hearing loss is a condition that can become extremely debilitating	2%	2%	9%	43%	44%	**4.25**
12. Hearing loss plays an important role in the acquisition of the speech and language of a child	2%	2%	8%	32%	57%	**4.40**
13. Suspicion of hearing loss is a strong reason for referral by an individual, parent, or caregiver	3%	3%	9%	51%	35%	**4.13**
14. It is essential to do hearing screening for newborns	2%	3%	12%	37%	47%	**4.23**
15. Whenever necessary, hearing aids can be fitted to children of any age	4%	11%	42%	23%	21%	**3.46**
16. Would like to receive additional information on hearing loss	3%	5%	17%	40%	35%	**4.01**
**Overall attitude**	**3%**	**4%**	**16%**	**38%**	**40%**	**4.08**

Abbreviations: A, agree; D, disagree; N, neutral; SA, strongly agree; SD, strongly disagree.

### Inferential Analysis

#### Correlation Analysis

Pearson's coefficient analysis measuring the correlation between knowledge of and attitudes toward hearing loss showed a positive and significant correlation at the 1% level (r = 0.817, *p* < 0.001).

#### Simple Regression Analysis

Knowledge of hearing loss (β = 0.817, t = 25.284, *p* < 0.001) significantly positively influenced attitudes toward hearing loss at the 1% level. This suggests that respondents' attitudes toward hearing loss were heavily influenced by their knowledge of hearing loss.

#### Cross-sectional Analysis

Both knowledge of and attitudes toward hearing loss were significantly associated with the type of physician, number of years of practice, and department at the 1% level, but not with practice setting. Significant associations were further investigated via nonparametric post-hoc tests as follows.

### Knowledge of Hearing Loss

Pairwise comparisons showed that medical interns had significantly more knowledge of hearing loss than general practitioners but significantly less than pediatricians and ENT specialists. Medical specialists had significantly more knowledge of hearing loss than general practitioners and other physicians but significantly less than pediatricians and ENT specialists.

As expected, physicians with at least ten years of practice had significantly more knowledge of hearing loss than those with less than 5 years or 5–9 years of practice.

Physicians working in medical departments had significantly more knowledge of hearing loss than those in casualty departments. Those working in pediatric departments had significantly more knowledge of hearing loss than those working in medical, other, and unsorted departments. Physicians working in ENT departments had significantly more knowledge of hearing loss than those working in medical, casualty, unsorted, and other departments.

### Attitudes Toward Hearing Loss

Medical interns had significantly poorer attitudes toward hearing loss than general practitioners, pediatricians, and ENT specialists. Moreover, medical specialists and general practitioners had significantly poorer attitudes toward hearing loss than pediatricians and ENT specialists.

Physicians with less than 5 years of practice had significantly poorer attitudes toward hearing loss than those with at least 10 years of practice. Physicians working in pediatric and ENT departments had significantly better attitudes toward hearing loss than those working in medical, casualty, unsorted, and other departments.

## Discussion

The results of this study shed light on the knowledge of and attitudes toward hearing loss among PCPs employed in the public health sector in Mauritius.

### Knowledge of Hearing Loss

Adequate knowledge of audiological management among PCPs is essential since it facilitates the early detection of hearing loss and timely intervention and improves auditory access to ensure maximum outcomes in speech and language development, especially in children. 1 A lack of knowledge among PCPs can perpetuate delays in identifying hearing loss and its diagnosis. 13 The two questionnaire statements that dealt with the knowledge of hearing loss in relation to its identification and intervention (items 5 and 6) showed mixed responses. Although 35% of respondents agreed that treatment exists to manage hearing loss, the same percentage of respondents were unaware of the specific management of hearing loss. Similarly, 32–33% either agreed or were unsure that hearing loss could be identified at any given age. One reason for the observed gap in knowledge of hearing loss among PCPs may be the lack of an early hearing detection and intervention program in the public health sector, such as a hearing screening program leading to unfamiliarity among PCPs about current practices related to managing individuals with hearing loss. Such gaps in knowledge among various disciplines of healthcare professionals have been reported in several studies. 17 18 19

A multiple-choice question (item 7) asked the PCPs to name the professional responsible for hearing assessments. This question was included to determine whether PCPs were aware of audiologists' vital role in the assessment of patients with hearing loss and subsequent intervention. This question also evaluated whether more knowledge is required regarding the role of audiologists. 20 The fact that most PCPs chose ENT specialists shows that PCPs were unaware of the role of audiologists. PCPs may need to be made aware of the presence of audiology departments that provide a variety of audiological services, including assessment, diagnosis, and therapeutic treatment of individuals with hearing loss. Thus, there is a need to spread awareness in Mauritius's public health sector about the role that audiologists play in diagnosing hearing loss and its intervention. This is critical to ensure that patients with hearing loss who seek services in the public health sector of Mauritius receive appropriate audiology services for timely diagnosis and prompt treatment. Without an appropriate referral to audiologists, the identification and management of hearing loss may be delayed 20 since patients that require hearing aids are provided with free behind-the-ear hearing aids by the Ministry of Social Security and National Solidarity, whereas individuals with hearing loss as a result of otological manifestations are monitored by the ENT specialists in the public health sector. 21

Physicians with any medical background should also possess adequate knowledge regarding the noninvasiveness of hearing tests. 22 This will allow physicians to guide families toward timely assessment and therapeutic intervention. Most PCPs (51.6%) responded correctly to this particular statement (item 8). This finding is encouraging because it means that PCPs in Mauritius will be able to correctly guide families when the situation arises.

PCPs responded diversely to the two open-ended statements (items 9 and 10) asking physicians to list all hearing tests they knew of to assess hearing sensitivity in children and adults, respectively. Otoacoustic emissions were the most popular response in children, followed by auditory brainstem responses and tuning forks. Otoacoustic emissions and auditory brainstem responses may have been the most popular choices for tests for many reasons. Otoacoustic emissions, which measure sound waves produced by outer hair cells in the cochlea are more well-known because they are a reliable and quick method, with a sensitivity of 100% and specificity of 98%. 23 Additionally, young children up to the age of 6 months cannot be tested using conventional subjective hearing tests, such as visual reinforcement and play audiometry, which are recommended tests for obtaining behavioral hearing thresholds in infants and children with a developmental age of more than 6 months. 23 Visual reinforcement audiometry is a subjective test that relies on the cooperation of the child, in which visual reinforcement is provided when the child responds to the presentation of a sound. 24 Play audiometry requires the child to perform an action such as placing a block in a container when he or she hears a sound. 24 In the study by Zaitoun et al. 25 most respondents thought that auditory brainstem response tests were more accurate than behavioral tests for establishing hearing thresholds in children. Yerranguntla et al. 14 reported that the most common response was pure tone audiometry followed by tuning fork tests and the auditory brainstem response test, which is an objective measurement of the auditory pathway that estimates hearing thresholds by testing synchronous neural function in individuals who are unable to tolerate traditional behavioral tests. 26

Tuning fork tests were the most popular answer in adults, followed by pure tone audiometry and other responses. Tuning fork tests are widely used by ENT specialists for diagnosing hearing loss in otological patients, whereas pure tone audiometry is regarded as the gold standard test for diagnosing hearing loss as it assesses the whole auditory pathway from the outer, middle, and inner ear to the brain to establish hearing thresholds. 27

### Attitudes Toward Hearing Loss

Attitudes are difficult to explore and measure. 13 The overall objective of the questions in section C was to explore two basic concepts: the attitudes of PCPs toward hearing loss and their inclination to favor early diagnosis and intervention. As expected, most PCPs strongly agreed that hearing loss is a debilitating condition and that hearing plays a vital role in the acquisition of speech and language of a child. The importance assigned by PCPs to identify hearing loss is paramount to ensure that they refer children with suspected hearing loss to audiology units. This may subsequently facilitate prompt diagnosis and interventions for hearing loss. After the identification and diagnosis of hearing loss in children, early intervention services including adjustment of amplification devices followed by speech and language therapy sessions for aural habilitation are provided in the speech therapy units of the public health sector to maximize the potential of the child with hearing loss.

Early detection of hearing loss in Mauritius is currently passive due to the absence of a national policy for a hearing screening program. Therefore, parental concern and observations about developmental delays are of utmost importance to identify hearing loss and intervene. The value that physicians assign to parental concerns about the child's hearing loss is equally essential to ensure early diagnosis and one of the worst attitudes that physicians may have is not to pay attention to parents' doubts or dismiss concerns about their child's hearing. 13 This item was explored in question 13. Most respondents agreed that suspicion of hearing loss by parents or caregivers was a strong enough reason for a hearing test referral. These findings are interesting because they suggest that PCPs in the public health sector of Mauritius recognize the vital role of parents or caregivers in the referral process for hearing assessments. Parents are more effective than professionals in identifying and diagnosing various health problems in their children. 28 Olusanya et al. 28 reported that parental suspicion of hearing loss in their children was the initial mode of detecting hearing loss. However, physicians who downplayed the parents' suspicion of hearing loss delayed the age of identification and intervention for hearing loss in these children.

Responses to the closely linked item 14 showed overwhelmingly positive responses in favor of newborn hearing screening. This was a positive finding, as it may indicate the readiness of PCPs to implement a hearing screening program. This also highlights the possibility of a collaborative approach between audiologists and physicians to successfully implement a program for early hearing detection and intervention. Therefore, awareness programs on hearing loss should be rolled out for PCPs in the public health sector of Mauritius to efficiently implement a newborn hearing screening program. By conducting hearing screenings, conditions that may otherwise result in delayed speech and language development, literacy, socialization, and school performance can be identified. 5

Auditory training and speech and language therapy are essential aspects of managing individuals with hearing loss. Question 15 investigated the attitude toward the need for hearing aids in children with hearing loss. In this question, a conditional clause (“whenever necessary hearing aids can be fitted to children of any age”) was intentionally included to facilitate the answer. 13 PCPs commonly ignore indications for the use of hearing aids. 13 Since PCPs are not meant to be experts on this specific matter, this question was presented as an attitude inquiry. 13 Most respondents reported being aware of the need for hearing aids for children of any age, whenever necessary. A likely reason for the high response to this question may be because children identified with HL in the public health sector of Mauritius are fitted with free behind-the-ear hearing aids and in December 2022 the first-ever cochlear implant surgery in the public health sector of Mauritius was performed on two children with profound hearing losses.

Question 16 aimed to identify any gaps in the existing knowledge that may influence the cooperation of PCPs in referring patients for hearing evaluations. 20 The answers indicated that PCPs would be interested in knowing more about hearing loss. Since PCPs as primary healthcare professionals guide patients and caregivers through the process of hearing evaluations, they need to have accurate knowledge of hearing loss. 6 13

Surprisingly, medical interns had greater knowledge of hearing loss than general physicians. General physicians in Mauritius have more hands-on clinical training experience than medical interns who may be more familiar with the theoretical aspects of hearing loss because they must undergo a pre-registration entry examination before their hospital internship. However, general physicians are required to be posted in various medical departments, such as orthopedic or surgical units, where they may not be required to possess adequate knowledge of hearing loss.

Regarding attitudes toward hearing loss, pediatricians and ENT specialists achieved significantly higher scores. The only study on hearing loss in Mauritius reported that when parents suspected hearing loss in their child, they contacted specialists such as ENT specialists and pediatricians in hospital services. 21 Pediatricians are professionals who parents visit for any difficulties with their child's health, whereas ENT specialists are the first and main sources of information about hearing loss. 25 The decision to undergo specific management after hearing assessment is also strongly supported by ENT specialists. 25 Therefore, both pediatricians and ENT specialists need to exhibit positive attitudes toward hearing loss since these physicians need to make appropriate referrals and support to individuals with hearing loss. 25 This was the case in this study.

Gaps in attitude levels among medical interns, general practitioners, and medical specialists may be attributed to the fact that they did not receive enough information about hearing loss in medical school. This highlights the need to formulate robust awareness courses on hearing loss among these healthcare professionals in the public health sector of Mauritius.

In clinical practice, the results of our study will be useful in determining the weaknesses and strengths of PCPs regarding their knowledge of and attitudes toward hearing loss. This knowledge can effectively highlight the areas of audiology that need to be considered for improving the knowledge of and attitudes toward hearing loss among PCPs in the public health sector of Mauritius.

### Strengths and Limitations

The present study's findings are unique because this was a novel attempt to examine the knowledge of and attitudes toward hearing loss in Mauritius. However, the analysis was based on self-reported data without validation of individual answers. The respondents may have been influenced by their colleagues' opinions. Additionally, the knowledge and attitudes among physicians who were not part of this study may differ. Therefore, future studies with a larger population-based sample are necessary to improve the generalizability of the results. This will enable more accurate and representative findings.

## Conclusion

The present study showed that PCPs demonstrated insufficient knowledge in some domains related to hearing loss, such as the early identification and intervention of hearing loss, professionals responsible for hearing assessments, and hearing assessments used for assessing hearing sensitivity. It also revealed generally positive attitudes toward hearing loss, such as the importance of hearing in the acquisition of speech and language development, the need for hearing screening in newborns, and the consideration of hearing loss suspected by parents. These findings highlight the importance of awareness programs tailored to improve the knowledge of hearing loss among PCPs in the public health sector of Mauritius. Physicians should be reminded of their critical and pivotal role in the early identification of hearing loss. This can be achieved by raising awareness of hearing loss through brochures and evidence-based short continuing medical education sessions, considering physicians' busy schedules. The essential aspect when educating physicians about hearing loss is to enable them to recognize potential hearing-related problems and when and how to refer patients for hearing tests. 7 This will ensure that PCPs actively guide patients toward a diagnosis of hearing loss, with the Ministry of Health and Wellness playing a vital role in this process.
