# Supplementary material for: Knowledge of and Attitudes Toward Hearing Loss Among Primary Care Physicians in the Public Health Sector of Mauritius
Source: Int Arch Otorhinolaryngol. 2023 Oct 6;28(2):e188–95. doi: 10.1055/s-0043-1770729 (PMC11008940; doi:10.1055/s-0043-1770729)
Supplement: Supplementary file 1 — Supplementary Material [file 10-1055-s-0043-1770729-s2023021485or.pdf]

Supplementary Appendix A: The Public Health Sector of Mauritius: Knowledge of and Attitudes Toward Hearing Loss Among Primary Care Physicians

Section A: Demographics

1. **Type of physician**
   ☐ General Practitioner ☐ Pediatrician
   ☐ ENT Specialist ☐ Medical Specialist
   Other specialty (please specify): ______________________

2. **What is your practice setting? [Tick all that apply]**
   ☐ Area Health Centre ☐ Community Health Centre ☐ Medi-clinic Hospital ☐ Other (please specify): _______________

3. **For how long (years) have you been in practice?**
   ☐ Less than 5 ☐ 5–9 ☐ 10 or more

4. **In which department are you currently based?**
   ☐ Pediatrics ☐ Neuro ☐ Casualty ☐ Medical ENT
   ☐ Other (please specify): ______________________

Section B: Knowledge of hearing loss

Please indicate the extent to which you agree with the following statements.
1 = Strongly disagree 2 = Disagree 3 = Neutral/Not sure 4 = Agree 5 = Strongly agree

	Questions	1	2	3	4	5
5.	Treatment exists to manage hearing loss	☐	☐	☐	☐	☐
6.	Hearing loss can be identified at any given age	☐	☐	☐	☐	☐

7. According to you, which professional(s) is/are responsible for hearing assessment? [Tick all that apply]
☐ General Practitioner ☐ ENT specialist
☐ Audiologist ☐ Other (please specify):

8. Hearing assessments (Select only one option)
☐ Are not invasive and can be performed at any age
☐ Are reliable and precise at the time the central nervous system reaches maturation
☐ Are invasive, but give precise information at any age

9. Please list all the hearing tests, which in your opinion, are used for assessing hearing sensitivity in newborns and children.

____
____

10. Please list all the hearing tests, which in your opinion, are used for assessing hearing sensitivity in adults.

____
____

 © 2023. Fundação Otorrinolaringologia. All rights reserved.

Section C: Attitudes toward hearing loss

	Questions	1	2	3	4	5
11.	Hearing loss is a condition that can become extremely debilitating	☐	☐	☐	☐	☐
12.	Hearing plays an important role in the acquisition of the speech and language of a child	☐	☐	☐	☐	☐
13.	Suspicion of hearing loss is a strong enough reason for referral by an individual, parent or caregiver	☐	☐	☐	☐	☐
14.	It is essential to do hearing screening for newborns	☐	☐	☐	☐	☐
15.	Whenever necessary, hearing aids can be fitted to children of at any age	☐	☐	☐	☐	☐
16.	Would like to receive additional information on hearing loss	☐	☐	☐	☐	☐

*** End of questionnaire ***
Thank you for your time and response.

 © 2023. Fundação Otorrinolaringologia. All rights reserved.
